# Supplementary material for: Following the long-term evolution of sp3-type defects in tritiated graphene using Raman spectroscopy
Source: Nanoscale Adv. 2026 Jan 16;8(4):1291–300. doi: 10.1039/d5na01051a (PMC12809252; doi:10.1039/d5na01051a)
Supplement: NA-008-D5NA01051A-s001 [file NA-008-D5NA01051A-s001.pdf]

Supplementary Information (SI) for Nanoscale Advances.
This journal is © The Royal Society of Chemistry 2026

Supplementary material

Following the Long-Term Evolution of sp³-type Defects in Tritiated Graphene using Raman Spectroscopy

G. Zeller [1,*], M. Schlösser [1] and H.H. Telle [2]

[1] Tritium Laboratory Karlsruhe (TLK), Institute for Astroparticle Physics (IAP), Karlsruhe Institute of Technology (KIT), Hermann-von-Helmholtz-Platz 1, 76344 Eggenstein-Leopoldshafen, Germany

[2] Departamento de Química Física Aplicada, Universidad Autónoma de Madrid, Campus Cantoblanco, 28049 Madrid, Spain

* Correspondence: genrich.zeller@kit.edu

Table of Content

page

S1 – CRM Raman spectral sensitivity calibration based on the NIST SRM 2242a standard 1

S2 – Representative Raman spectra for all samples, with Lorentzian fit to Raman features 2

S3 – Information extracted from the Raman spectroscopic maps of the samples 3
  S3.1 – Numerical values for all Raman map points, and their graphical representation 5
  S3.2 – Concept for the determination of the concentrations of defect types 6
  S3.3 – Example calculation 7

References 8

S1 – CRM Raman spectral sensitivity calibration based on the NIST SRM 2242a standard

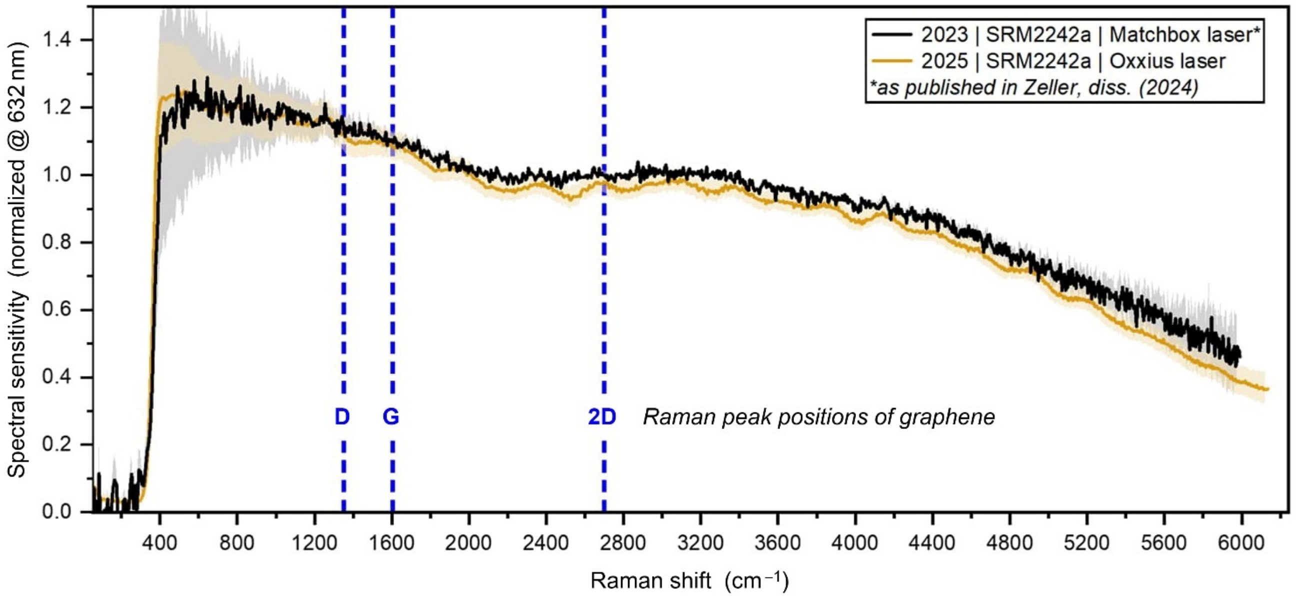

Figure S1: Long-term stability and comparability of the Raman system, based on response measurements using the NIST SRM 2242a standard.[39] The sensitivity curves for different years and hardware configurations (including new gratings and a replaced laser) agree within 1σ uncertainties, demonstrating year-over-year cross-comparability. The curves are normalized to =1 at the wavelength of the fluorescence emission maximum of the standard.

S2 – Representative Raman spectra for all samples, with Voigt fits to Raman features

For the fit procedure, pseudo-Voigt profile functions were utilised.[40,41] In an often-used mathematical description they are given as the linear combination

$$V(x;\mathrm{w}_{(L)},\mathrm{w}_{(G)}) = \eta(r)\cdot L_V(x;\mathrm{w}_{(V)}) + (1-\eta(r))\cdot G_V(x;\mathrm{w}_{(V)}) \;, \quad (1)$$

Its two common parameters are

- the width parameter $\mathrm{FWHM}_{(V)} \equiv \mathrm{w}_{(V)} \approx 0.5346\cdot\mathrm{w}_{(L)} + \sqrt{\mathrm{w}_{(G)} + 0.2166\cdot\mathrm{w}_{(L)}^2}$, and (2)
- the mixing parameter $\eta(r) = 1.36603\cdot r - 0.47719\cdot r^2 + 0.11116\cdot r^3$, with $r \equiv \frac{\mathrm{w}_{(L)}}{\mathrm{w}_{(V)}}$. (3)

Note that, in our fit implementation, the Lorentz (L) and Gaussian (G) component functions are taken in unit-height form and share the common width parameter, $\mathrm{w}_{(V)}$; thus, these line-shape functions are defined as

$$L(x;\mathrm{w}_{(V)}) = \frac{\mathrm{w}_{(V)}^2}{4\cdot(x-x_c)^2 + \mathrm{w}_{(V)}^2} \quad \text{and} \quad G(x;\mathrm{w}_{(V)}) = \exp\left(-\frac{(x-x_c)^2}{2\sigma_{(G)}^2}\right). \quad (4)$$

Here the Gaussian component of $V(x;\mathrm{w}_{(L)},\mathrm{w}_{(G)})$ is associated with the resolution of the spectrometer, whose $\mathrm{FWHM}_{(G)}$ is evaluated from the analysis of the atomic line emission from a Ne-lamp (used for wavelength calibration of the spectrometer). For the spectrometer configuration used in campaign 0 this was $\mathrm{FWHM}_{(G)}(0) = 32 \pm 4\ \mathrm{cm}^{-1}$, while for campaigns I and II it was $\mathrm{FWHM}_{(G)}(\mathrm{I,II}) = 9.6 \pm 0.7\ \mathrm{cm}^{-1}$.

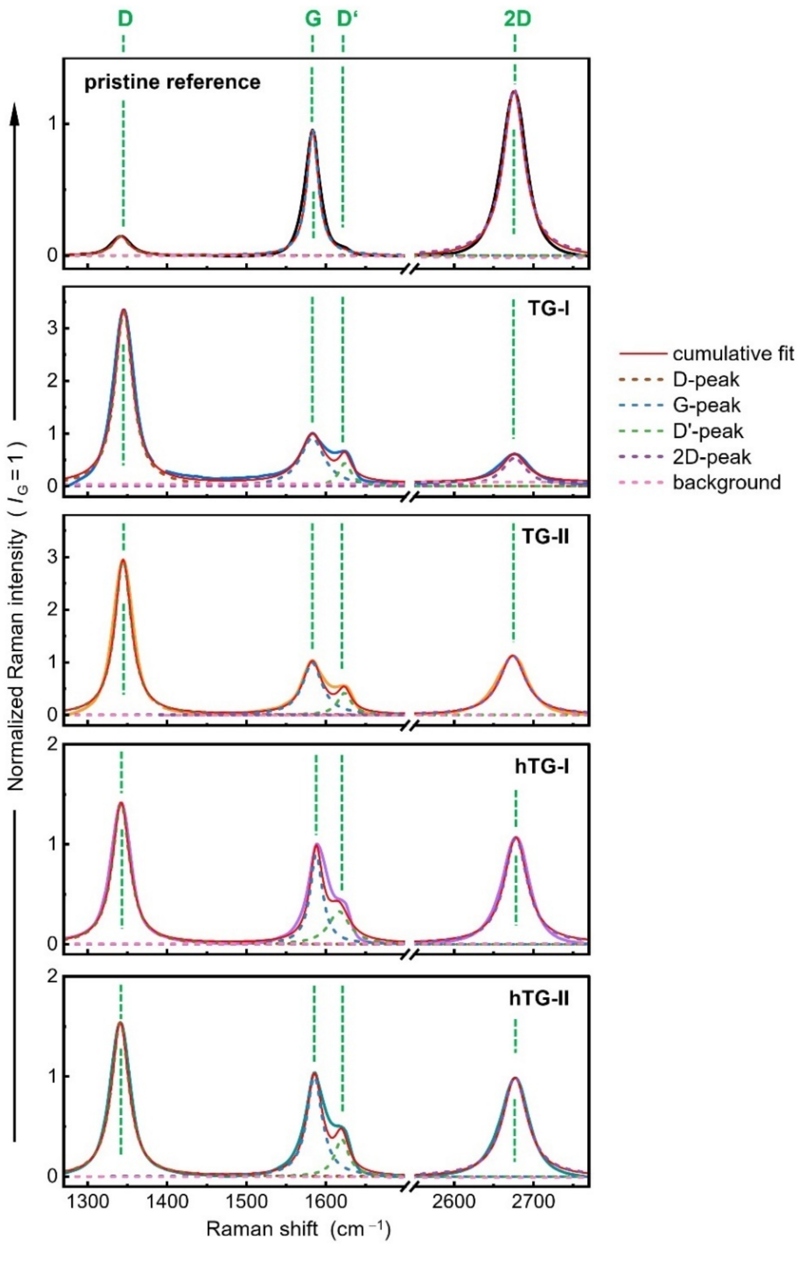

Figure S2: Representative Raman spectra of all sample data sets (pristine, TG-I, TG-II, hTG-I and hTG-II), shown with individual Voigt peak-fits and cumulative fit results. TG = tritiated graphene; hTG = heated tritiated graphene.

S3 – Information extracted from the Raman spectroscopic maps of the samples

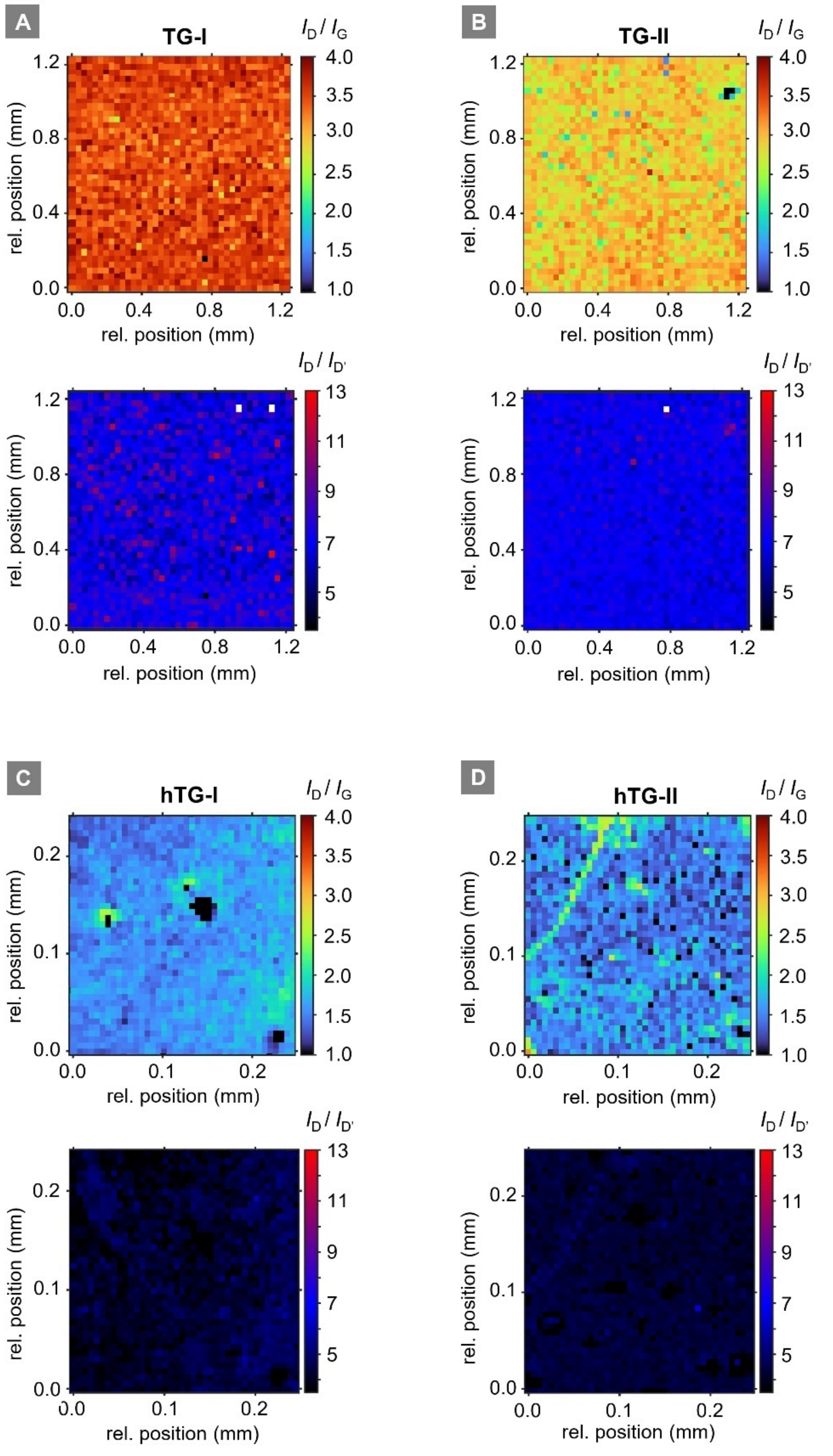

Figure S3: Raman spectroscopic maps of all graphene samples. Note that the one-year-later maps were not recorded for exactly equal areas of the samples. Top rows: I_D/I_G intensity ratios; bottom rows: $I_D/I_{D'}$ intensity ratios.

(A) 1.2 × 1.2 mm^2 area scan with step size of ΔS = 32 µm of T_2-exposed graphene.

(B) 1.2 × 1.2 mm^2 area scan with step size of ΔS = 32 µm of T_2-exposed graphene, after one year of storage in laboratory atmosphere.

(C) 0.25 × 0.25 mm^2 area scan with step size of ΔS = 7 µm of T_2-exposed graphene, heated at 500 °C for 24 h.

(D) 0.25 × 0.25 mm^2 area scan with step size of ΔS = 7 µm of heated T_2-exposed graphene, after one year of storage in laboratory atmosphere.

S3.1 Numerical values for all Raman map points, and their graphical representation

From the line ratio maps for the D-, D'- and G-peaks displayed in Figure S3 one can extract the actual numerical values, and plot these in the form I_D / I_G versus $I_D / I_{D'}$. This plot is shown in Figure S4. Note that Figure 4 in the main text is a simplified representation of these data, in the form of a contour plot, lumping data points together around the mean (as 1σ-, 2σ- and 3σ contours regions).

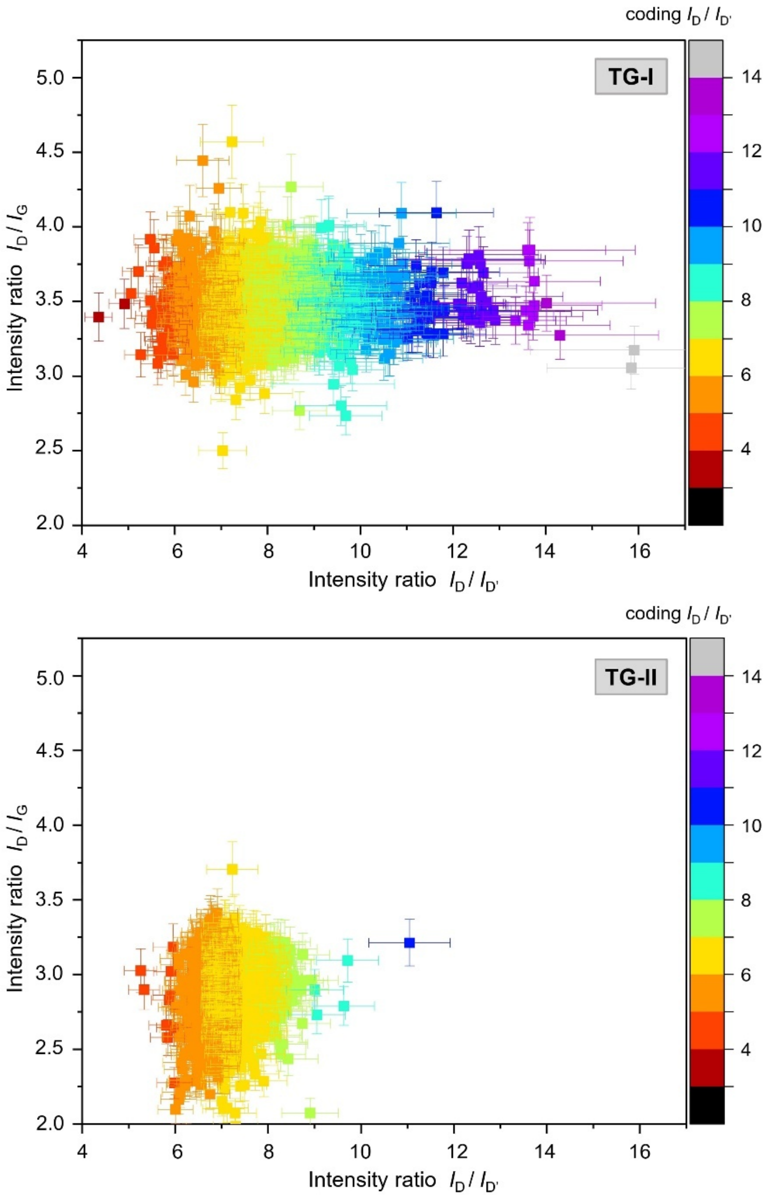

Figure S4: Intensity ratio plots of I_D / I_G versus $I_D / I_{D'}$ of the 40×40 data points from the Raman maps in Figures S3(a) and S3(b), including error bars for each individual data point.

Note that in the figure the D/D' classification values are colour-coded according to integer-value ranges (analogous to the defect-type limiters).

For classification and interpretation, the values for each integer range are summed up, and the sum-values are shown as relative fractions of the total in Figure 6 of the main text. Said diagram serves as a visualisation aid in the discussion of the defect-type densities.

All (mean) evaluation parameters used for the presentation and interpretation of the experimental data – i.e., numerical values (including errors) for peak position, peak width and intensity ratios for the relevant Raman features G, D, D' and 2D – are summarised in Table S1.

Table S1: Mean fit results for all data sets. Given uncertainties are calculated from individual per spectra uncertainties, $\bar{u}_i$, and spread of the data points, s: $\sigma_{\mathrm{tot}}=(\bar{u}_i^2+s^2)^{1/2}$. Note that, the individual uncertainties are much smaller than the spread of the data points within each data set, therefore, in first order, one can approximate $\sigma_{\mathrm{tot}}\approx s$. The mixing parameter is defined in equation (4) of S2.

	Peaks	Pristine	TG-I	TG-II	hTG-I	hTG-II
**Intensity ratio**	D/G	0.12 ± 0.01	3.51 ± 0.26	2.90 ± 0.28	1.60 ± 0.24	1.53 ± 0.29
	D'/G	0.02 ± 0.01	0.49 ± 0.07	0.42 ± 0.05	0.38 ± 0.06	0.37 ± 0.11
	D/D'	6.22 ± 1.50	7.18 ± 1.00	6.94 ± 0.56	4.22 ± 0.50	4.26 ± 0.32
	2D / G	1.75 ± 0.10	0.70 ± 0.09	1.28 ± 0.27	1.36 ± 1.08	1.15 ± 0.16
	D / 2D	0.07 ± 0.01	5.07 ± 0.57	2.32 ± 0.37	1.22 ± 0.31	1.41 ± 0.71
**FWHM*(L)* in cm^{-1}**	D (w_D)	54.2 ± 5.5	28.8 ± 1.3	27.4 ± 1.2	29.3 ± 3.5	31.8 ± 7.1
	G (w_G)	16.3 ± 0.9	39.7 ± 6.2	33.8 ± 3.4	21.3 ± 4.0	26.5 ± 6.0
	D' ($w_{D'}$)	21.8 ± 17.5	21.1 ± 6.5	22.4 ± 2.7	32.8 ± 8.7	23.6 ± 3.6
	2D (w_{2D})	32.9 ± 0.7	41.6 ± 6.6	44.8 ± 6.7	37.4 ± 4.3	39.0 ± 6.0
**Position in cm^{-1}**	D	1344.7 ± 1.2	1344.5 ± 3.3	1342.9 ± 0.8	1329.3 ± 1.3	1339.6 ± 4.7
	G	1584.5 ± 0.9	1581.9 ± 3.9	1581.0 ± 1.3	1572.6 ± 1.5	1583.4 ± 5.6
	D'	1621.1 ± 5.3	1622.7 ± 4.0	1621.8 ± 1.3	1601.3 ± 1.5	1618.7 ± 5.7
	2D	2677.0 ± 1.8	2674.0 ± 6.7	2672.7 ± 1.6	2652.2 ± 2.6	2673.8 ± 20.3
**Mixing parameter η**	D	0.98 ± 0.01	0.93 ± 0.02	0.92 ± 0.01	0.92 ± 0.09	0.93 ± 0.03
	G	0.83 ± 0.01	0.95 ± 0.03	0.94 ± 0.01	0.87 ± 0.09	0.91 ± 0.04
	D'	0.83 ± 0.11	0.86 ± 0.07	0.89 ± 0.02	0.92 ± 0.10	0.89 ± 0.04
	2D	0.94 ± 0.01	0.96 ± 0.03	0.97 ± 0.00	0.94 ± 0.09	0.95 ± 0.04

S3.2 Concept for the determination of the concentrations of defect types

By and large, defect types (x) may be separated into two broad classes, namely adsorption-type (x≡a) and vacancy-type (x≡v) defects. According to established, descriptive models for defects in graphene they manifest themselves in changes in the amplitude and shape of Raman spectral features. For stage-1 defect-density samples, i.e., samples with small to moderate defect densities, one can take away two key messages (based on said models):

(i) the intensity ratio I_D/I_G is proportional to the defect density, L_D(x), in units of (nm); and

(ii) the intensity ratio $I_D/I_{D'}$ yields distinct associations: $I_D/I_{D'} \geq 13$ is indicative for adsorption defects; $I_D/I_{D'} \leq 7$ for vacancy defects; and $I_D/I_{D'}$ in the range 7⋯13 for mixed defects.

Note however that, mixing cannot necessarily be assumed to be linear.

The established model for vacancy-type defects is that by Lucchese and co-workers,[32] which was generalized for different laser excitation energies, E_L (in eV), by Cançado and co-workers.[33] The latter describes the relationship between I_D/I_G and $L_D(\mathrm{v})$ as

$$\frac{I_D}{I_G}(L_D) = C_A \frac{r_A^2-r_S^2}{r_A^2-2r_S^2}\cdot\left[e^{-\pi\frac{r_S^2}{L_D^2}} - e^{-\pi\frac{r_A^2-r_S^2}{L_D^2}}\right] + C_S\left[1-e^{-\pi\frac{r_S^2}{L_D^2}}\right] \quad (5)$$

with $C_A = 160\cdot E_L^{-4}\cdot \mathrm{eV}^{-4}$, $C_S = 0.87 \pm 0.05$, $r_A = (3.00 \pm 0.03)\mathrm{nm}$, and $r_S = (1.00 \pm 0.04)\mathrm{nm}$.

Recently Fournier and co-workers published a model specifically tailored for sp[3]-type defects.[36] There the relationship between I_D/I_G and $L_D(\mathrm{a})$ is defined by

$$\frac{I_D}{I_G}(L_D) = \frac{C_S f_S(L_D)+C_A f_A(L_D)}{1-f_{sp^3}(L_D)} , \quad (6)$$

where

$$f_A(L_D) = e^{-\pi\frac{r_S^2}{L_D^2}} - e^{-\pi\frac{r_A^2}{L_D^2}} ,\; f_S(L_D) = e^{-\pi\frac{r_{sp^3}^2}{L_D^2}} - e^{-\pi\frac{r_S^2}{L_D^2}} \text{ and } f_{sp^3}(L_D) = 1 - e^{-\pi\frac{r_{sp^3}^2}{L_D^2}} , \quad (7)$$

and $C_A = 20.1$, $C_S = 0.86$, $r_A = 0.242\ \mathrm{nm}$, $r_S = 0.183\ \mathrm{nm}$ and $r_{sp^3} = 0.0913\ \mathrm{nm}$.

When evaluating and comparing our data – using these two models – one finds that, the Raman response to the two defect types is significantly more sensitive for vacancy-type defects than for adsorption-type defects. For example, a value of $I_D / I_G = 0.1$ would correspond to

L_D(Lucchese / Cançado) = 37.1 nm , or

L_D(Fournier) = 4.1 nm .

This means that, when quantitatively comparing data sets, for compositional changes, both variations in I_D / I_G and $I_D / I_{D'}$ need to be tracked in parallel.

S3.3 Example calculation

Simplified versions of the complex equations (1) and (2) can be found in the literature, which allow for an analytical inversion; however, these are in general only applicable in the low-defect density regime. Since this is not the case for the samples studied in this work, we use a different approach.

In order to accurately evaluate the defect density, L_D, from the intensity ratio, I_D / I_G, we calculate the correlation curves according to the two models, and then perform numerical interpolation. This principle is visualised for the TG-I data in Figure S5.

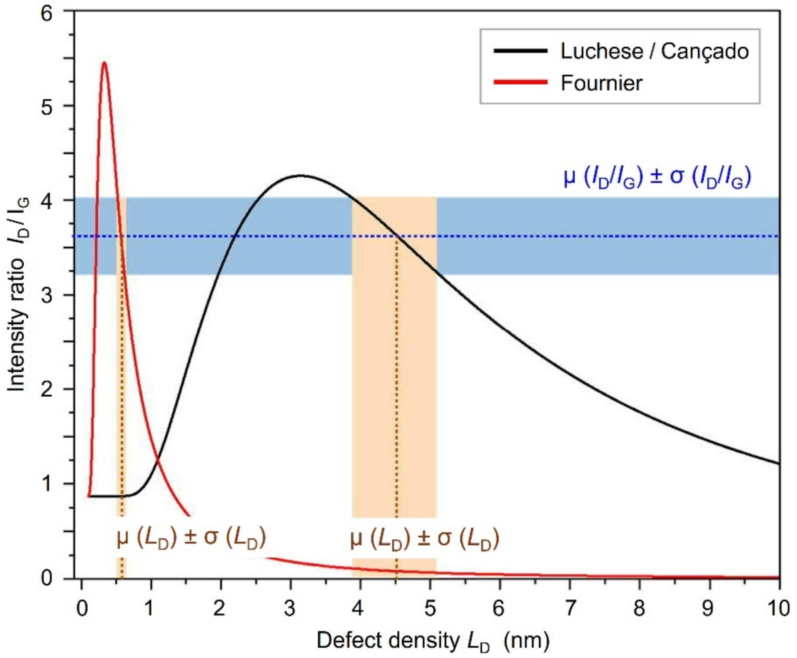

Figure S5: Calculated numerical I_D/I_G vs L_D curves, according to the models of Lucchese/Cançado and Fournier. The blue dotted line and the blue band correspond to the mean I_D/I_G with 1σ standard uncertainty of the TG-I data set. As it is shown in the main document, the TG-I data fall to the low-defect regime (Stage 1); therefore, the crossing as smaller L_D-values is correct here. The orange lines and bands visualise the L_D-values and their uncertainties; they are obtained by numerically interpolating the intersection points with the blue line, and their uncertainty, for each of the two model curves.

References

32 M.M. Lucchese, F. Stavale, E.H. Martins Ferreira, C. Vilani, M.V.O. Moutinho, R.B. Capaz, C.A. Achete, A. Jorio, Quantifying ion-induced defects and Raman relaxation length in graphene, *Carbon* 2010, **48** (5), 1592-1597. https://doi.org/10.1016/j.carbon.2009.12.057

33 L. G. Cançado, A. Jorio, E. H. M. Ferreira, F. Stavale, C. A. Achete, R. B. Capaz, M. V. O. Moutinho, A. Lombardo, T. S. Kulmala and A. C. Ferrari, Quantifying defects in graphene via Raman spectroscopy at different excitation energies, *Nano Lett*, 2011, **11**, 3190–3196. https://doi.org/10.1016/j.carbon.2024.118801

36 T. Fournier, C. Crespos, I. Arshad, M. Dubois, B. Lassagne, M. Monthioux, F. Piazza and P. Puech, Quantifying the sp^3/sp^2 ratio in functionalized graphene, *Carbon*, 2025, **244**, 120657. https://doi.org/10.1016/j.carbon.2025.120657

39 C.A. Gonzalez and S.J. Choquette; Chemical Sciences Division Standard Reference Material® 2242a Relative Intensity Correction Standard for Raman Spectroscopy: 532 nm Excitation, Certificate, Issue 20 June 2025. National Institute of Standards and Technology (NIST), Gaithersburg (MD), USA. https://tsapps.nist.gov/srmext/certificates/2242a.pdf [accessed November 6, 2025]

40 J.J. Olivero and R.L. Longbothum, Empirical fits to the Voigt line width: A brief review, *JQSRT*, 1977, **17** (2), 233-236.

41 J. Rodríguez-Carvajal, *FullProf Manual*, Institut Laue–Langevin (ILL), Grenoble, France, 2001. https://www.ill.eu/sites/fullprof/downloads/Docs/FullProf_Manual.pdf [accessed November 6, 2025]
